# Population pharmacokinetics of the humanised monoclonal antibody, HuHMFG1 (AS1402), derived from a phase I study on breast cancer

**DOI:** 10.1038/sj.bjc.6605560

**Published:** 2010-02-16

**Authors:** B Royer, W Yin, M Pegram, N Ibrahim, C Villanueva, D Mir, F Erlandsson, X Pivot

**Affiliations:** 1 CHU Besançon, Laboratoire de Pharmacologie Clinique, Hôpital Jean Minjoz, Besançon 25030, France; 2 INSERM, UMR645, Besançon 25020, France; 3 Université de Franche-Comté, IFR133, Besançon, France; 4 EFS Bourgogne Franche-Comté, Besançon, France; 5 CIC intégré en Biothérapies 506, CHU Besançon, France; 6 Antisoma Research Limited, 566 Chiswick High Road, Chiswick, London W4 5YF, UK; 7 University of Miami Miller School of Medicine, 1475 NW 12th Avenue, Miami, FL 33136, USA; 8 MD Anderson Cancer Center, 1515 Holcombe Blvd, Houston, TX 77030, USA; 9 CHU Besançon, Service d’oncologie, Hôpital Jean Minjoz, Besançon 25030, France

**Keywords:** HuHMFG1, AS1402, population pharmacokinetic, antibody, linear two-compartment, breast cancer

## Abstract

**Background:**

HuHMFG1 (AS1402) is a humanised monoclonal antibody that has undergone a phase I trial in metastatic breast cancer. The aim of this study was to characterise the pharmacokinetics (PKs) of HuHMFG1 using a population PK model.

**Method:**

Data were derived from a phase I study of 26 patients receiving HuHMFG1 at doses ranging from 1 to 16 mg kg^−1^. Data were analysed using NONMEM software and covariates were included. A limited sampling strategy (LSS) was developed using training and a validation data set.

**Results:**

A linear two-compartment model was shown to be adequate to describe data. Covariate analysis indicated that weight was not related to clearance. An LSS was successfully developed on the basis of the model, in which one sample is collected immediately before the start of an infusion and the second is taken at the end of infusion.

**Conclusion:**

A two-compartment population PK model successfully describes HuHMFG1 behaviour. The model suggests using a fixed dose of HuHMFG1, which would simplify dosing. The model could be used to optimise dose level and dosing schedule if more data on the correlation between exposure and efficacy become available from future studies. The derived LSS could optimise further PK assessment of this antibody.

Breast cancer accounts for ∼25% of malignancies that affect women (Kumle, 2008). Metastatic breast cancer remains incurable and there is a need for new active treatments in this setting. HuHMFG1 is a recombinant DNA-derived humanised monoclonal human milk fat globule-1 antibody that targets the immunodominant epitope of the *MUC1* gene product (Pericleous *et al*, 2005). It was engineered by grafting the complementary determining regions of the parental murine antibody (HMFG1) into the consensus framework of a human IgG1. Unlike the low expression rate of antigens such as HER2 (20–25%), polymorphic epithelial mucin or MUC1 expression is considered to be universal (>90%) in breast cancer, as well as in other common epithelial cancers (Singh and Bandyopadhyay, 2007).

Monoclonal antibodies specific for tumour-associated antigens can induce an immunological cellular attack on tumour cells by a process known as antibody-dependent cell-mediated cytotoxicity (ADCC). HuHMFG1 has been demonstrated to be a potent mediator of ADCC by recruiting natural components of the body's immune system (Snijdewint *et al*, 2001) HuHMFG1 binds to the extracellular MUC1 peptide sequence, PDTR. These sequences are not exposed in normal cells because of full glycosylation, but aberrant glycosylation in cancer cells exposes the epitope to the antibody. Therefore, HuHMFG1 has the potential for targeted anti-cancer therapy in a wide range of MUC1 overexpressing epithelial tumours, including breast cancer. HuHMFG1 has entered a randomised phase II study in first-line ER+/HER2− metastatic breast cancer in combination with letrozole therapy.

Population pharmacokinetic (PK) models help to define the optimal schedule of drug administration and offer several benefits, including accounting for inter-subject variability through an assessment of covariates. This can improve the model and understanding of PK. Moreover, using a preferred model, a limited sampling strategy (LSS) can be created to undertake Bayesian estimation of individual PK parameters with the minimal number of patient samples (Royer *et al*, 2009). We present the results of a population PK model for HuHMFG1, together with a proposed LSS.

## Materials and methods

### Study design and patients

Data were derived from an open-label, non-randomised, dose-escalation phase I study (Pegram *et al*, 2009). The phase I study aimed to determine the safety, tolerability, PK and anti-tumour activity of HuHMFG1 monotherapy in patients with locally advanced or metastatic breast cancer who had previously been treated with up to three chemotherapeutic regimens, including neoadjuvant and adjuvant therapy. The following doses were studied: 1, 3, 9 and 16 mg kg^−1^. The study was conducted in accordance with the principles of the Declaration of Helsinki, and written informed consent was obtained from all patients before any study-specific screening procedures were performed. All patients had a PK assessment during their exposure to treatment and for up to 6 months after discontinuation of treatment.

### Drug administration and sampling

HuHMFG1 was administered as an intravenous infusion of 60–180 min using a rate-controlled infusion pump. The drug was dispensed into 250 ml normal saline (0.9% sodium chloride) infusion bags. The duration of infusion depended on the dose level: 60 min infusion at 1–3 mg kg^−1^; 120 min infusion at 9 mg kg^−1^; and 180 min infusion at 16 mg kg^−1^. Sampling was performed depending on the administered dose, as described in Table 1. At each time point, an aliquot of blood (3.5 ml) was collected from each patient using the opposite arm to that used for administration of the drug. Blood was collected in a serum separator II tube and immediately centrifuged at 1200 *g* (or 3000 r.p.m.) for 5 min at 4°C. Equal volumes of serum were transferred into two transfer tubes and stored at −20°C pending analysis.

### Drug assay

HuHMFG1 concentration was determined in human serum samples by means of an enzyme-linked immunosorbent assay in microtitre plate format. Calibration was carried out by performing a four-parameter fit (absorbance *vs* nominal concentration of calibration samples, including ‘0’ standard). The calibration range was 0–10.00 mg l^−1^. The lower limit of quantification for this assay was determined to be 0.50 mg l^−1^. Samples with measured concentration above the upper limit of quantification were re-analysed at a higher dilution.

### Population PK analysis

Pharmacokinetic data were analysed using the non-linear mixed effects modelling approach as implemented in NONMEM software version VI, level 1.0 (ICON Development Solutions, Ellicott City, MD, USA; Beal *et al*, 1989–2006). First-order conditional estimation with the INTERACTION option was used. Several models were investigated: one-, two- or three-compartmental linear models, with or without additional non-linear elimination. The choice between models was made by evaluation of goodness-of-fit. This assessment consisted of comparison of the following graphs: observed concentrations *vs* predictions (OBS–PRED) and weighted residuals *vs* predictions (WRES–PRED) using the R program.

Several models were investigated for residual variability: exponential, additive or a combination of both error models. Inter-individual variability was modelled with an exponential random effect.

The following covariates were investigated on V1 (central volume of distribution) and CL (the clearance), but not on V2 (peripheral volume) or Q (inter-compartmental clearance), for which no inter-subject variability could be isolated: age, body weight, height, body mass index, serum albumin, serum total protein concentration, creatinine clearance (Cockcroft and Gault, 1976), alkaline phosphatase (ALP), alanine aminotransferase (ALT), aspartate aminotransferase (AST), *γ*-glutamyl transpeptidase (GGT), CA15–3 and CA27.29 antigens, and the presence of liver metastasis. Human anti-human antibodies (HAHAs) were, at the minimum, to be assessed in all patients, irrespective of dose. Results of HAHA assessments were available from baseline and 4 weeks after start of therapy for 23 patients, and at 4 weeks after treatment discontinuation for 17 patients out of 26 enrolled patients. Between three and four HAHA assessment results were available for the majority of patients. The HAHA antibodies were not detected in the serum of any patients and thus not further investigated. Covariates were selected in the final population model if their effect was biologically plausible; if they produced a minimum reduction of 4 in the objective function value; if they produced a reduction in the variability of the PK parameter; and, finally, if a minimum increase of 7 was observed after removal from the final model.

The accuracy and robustness of the final models were evaluated by a bootstrap approach consisting of repeated random sampling with replacement from the original data using Wings for NONMEM (Holford *et al*, 1993a, 1993b; Sheiner, 1997). Re-sampling was performed 1000 times. Mean values and the precision of parameters obtained by this procedure were compared with those obtained with the original set. The final models were also evaluated using a visual predictive check (VPC) assessment obtained after 1000 simulations of the data set. The percentage of observed data outside the 5th and 95th percentiles of simulated concentrations was calculated to assess the final models. Together with these assessments, a normalised prediction distribution error (NPDE) assessment (Brendel *et al*, 2006) was performed. A total of 1000 Monte Carlo simulations were performed to calculate NPDE using an add-on package for R (Comets *et al*, 2008). The distribution of the obtained NPDE was compared with a normalised distribution.

An LSS was built with the aim of decreasing the number of samples required in further studies. The LSS was based on clearance prediction and performed by splitting patient results into two groups of 13 patients: one group for development of the LSS model and one group for validation and assessment of the model. The accuracy of the LSS model was assessed by computing the mean relative prediction error (mpe%). Precision of the model was assessed by the root mean squared relative prediction error (rmse%). These parameters were calculated as follows, where *N* is the number of patients and pej is the prediction error in the jth individual:
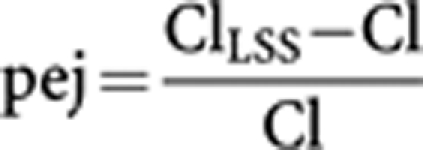


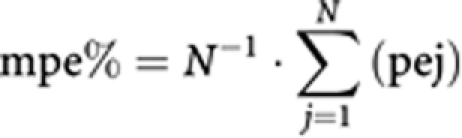


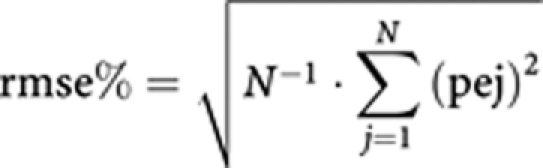
 The choice of times for the retained LSS was determined on the basis of the values of mpe% and rmse% and the convenience of sampling times.

## Results

### Patient population

**Figure 1:**
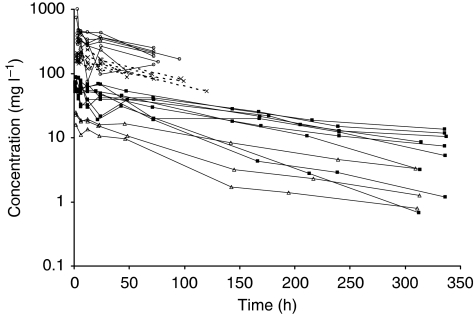
Semi-logarithmic representation of concentration–time profiles obtained from 26 patients during first administration of HuHMFG1. Administered doses were 1 mg kg^−1^ (white triangle, solid line), 3 mg kg^−1^ (black square, solid line), 9 mg kg^−1^ (cross, dashed line) and 16 mg kg^−1^ (open circle, solid line).

A total of 435 samples obtained from 26 patients were available for population PK analysis. The demographic characteristics of patients is summarised in Table 2. There were three, nine, six and eight patients in the 1, 3, 9 and 16 mg kg^−1^ groups, respectively. Data observed during the first administration are shown in Figure 1. In all, 24 patients received a second administration, 23 a third, 19 a fourth, 13 a fifth, 12 a sixth, 4 a seventh and 1 patient received 10 administrations.

### Population PK model

HuHMFG1 concentrations in the serum were best described by a two-compartment linear model with a zero-order infusion (ADVAN3 TRANS4 subroutine). The PK parameters calculated with this model were clearance (CL), central volume of distribution (V1), inter-compartmental clearance (Q) and peripheral volume of distribution (V2) (Table 3). Inter-patient variability was described by an exponential error model, whereas residual variability was described by a combined proportional and fixed additive error model. Inter-occasion variability was assessed with an exponential random effect and was found to be insignificant. Random effects could not be obtained for either Q or V2.

Among the tested covariates, ALP, GGT and AST lead to a significant decrease in the objective function of central CL only. Nonetheless, only AST was retained after the removal step, leading to a 32.4% decrease in associated variability. The final model including covariate is as follows, where AST_med_ is the median value of AST in the studied population:
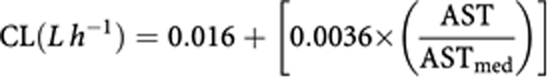


Body weight was not found to be a covariate, indicating that fixed dosing of HuHMFG1 may be a viable option.

**Figure 2:**
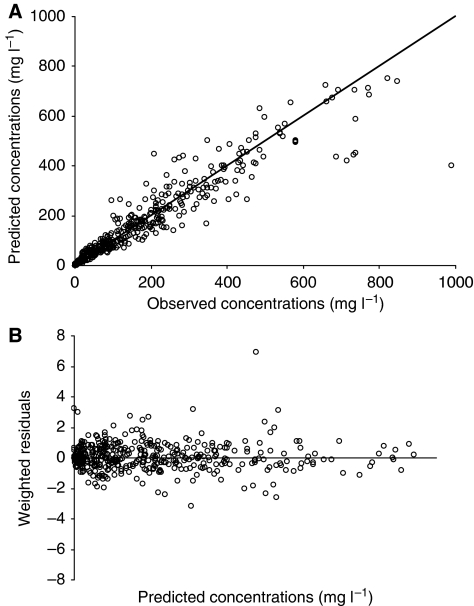
Goodness-of-fit obtained with the model objectified through observed concentrations *vs* (**A**) predicted (PRED) observations and through (**B**) weighted residuals (WRES) *vs* predicted (PRED) observations.

The assessment of goodness-of-fit of predicted *vs* observed HuHMFG1 concentrations and of weighted residuals *vs* predicted serum concentration for the final model is shown in Figure 2A and B. Bootstrap evaluation showed similar estimates compared with the original PK parameters, as shown in Table 3. A median distribution half-life of 1.87 (0.49–2.29) days and a terminal elimination half-life of 11.04 (4.38–15.04) days were further calculated from these parameters.

### Assessment of the model

**Figure 3:**
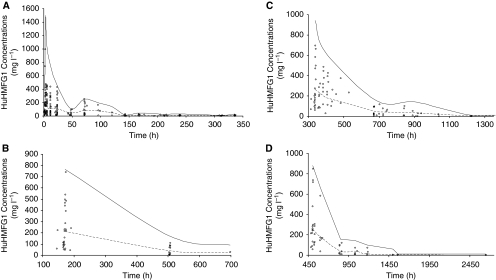
Accuracy of the final model evaluated by posterior visual predictive check assessment obtained after 1000 simulations. (**A**–**D**, respectively) Data correspond to the first four administrations, which are representative of further administrations. Solid lines correspond to 5th and 95th percentiles; dashed line corresponds to the median.

Evaluation of the model was undertaken using VPC assessment. The results of the first four administrations are shown in Figure 3A–D. One can observe that the limit of the 95th percentile could overestimate concentrations during the first 4 h after the first administration (Figure 3A). This could be due to the fact that, during this period, data include concentrations obtained from the end of infusion with high doses (9 and 16 mg kg^−1^) and concentrations at the declining phase after the end of infusion with low doses (1 and 3 mg kg^−1^) (Table 1). The combination of very high concentrations (e.g., from the end of infusion samples after a 16 mg kg^−1^ dose) and very low concentrations (e.g., from post-infusion samples after 1 mg kg^−1^ dose) could account for the observed discrepancy. When samples of the same dose range were separated at ∼48 h for samples from the 1 and 3 mg kg^−1^ cohorts, or at ∼72 h from the 9 and 16 mg kg^−1^ cohorts, the model described the concentrations successfully. The same conclusion could be drawn for the second, third and fourth administrations (Figure 3B–D). In every case, the model successfully described the CL of patients. Examination of the VPC figures and the WRES *vs* time graph for cycle 5–cycle 10 (data not shown) indicated that no bias appeared with time. Hence, even if the number of patients and samples was lower compared with that of previous cycles, on can draw similar conclusions for the fifth and later cycles.

**Figure 4:**
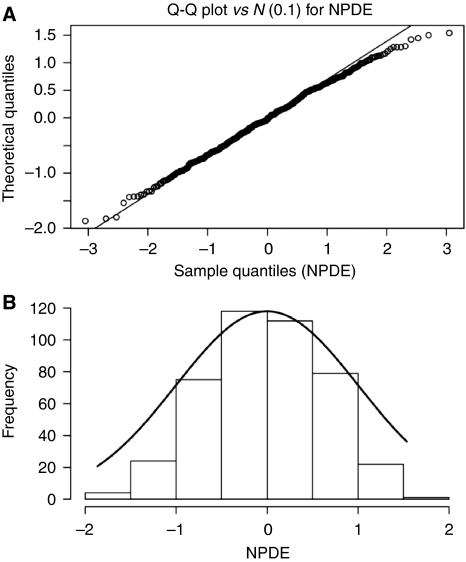
(**A**) Q–Q plot of the NPDE obtained after 1000 Monte Carlo simulations of the model. The solid line represents the identity line. (**B**) Shows the frequency distribution of the NPDE (histograms) compared with the theoretical normal distribution (solid line).

The model was further evaluated using the recently published NPDE assessment (Brendel *et al*, 2006). According to this assessment, when data are adequately described by the model, the calculated NPDE (performed after 1000 simulations) should follow a normal distribution. The distribution of NPDE obtained with the present model does follow a normal distribution, as observed in the Q–Q plot of Figure 4A and in the distribution graph of Figure 4B.

### Limited sampling strategy

To facilitate PK studies and to decrease the number of blood samples required for further studies, an LSS based on CL assessment was developed. Eight strategies were assessed with 1, 2 or 3 samples. Results obtained with these strategies using the training group are presented in Table 4. Taking into account accuracy (mpe%), precision (rmse%), number of samples and convenience of sampling, the LSS consisting of samples collected before and after infusion was retained. With this LSS, the validation group led to a similar mpe% of −1.41% and rmse% of 6.60%. Performing this LSS with all patients led to an mpe% of 3.23% and an rmse% of 7.74%. A correlation was also observed between actual CL values and predicted CL values, with a slope of 1.00 and an *r*^2^ of 0.913.

## Discussion and conclusion

This study establishes a population PK model for HuHMFG1, a recombinant DNA-derived humanised monoclonal antibody that targets the MUC1 gene product. The selected model is a two-compartmental model with linear elimination, and was shown to successfully describe the data using bootstrap, VPC and NPDE assessments. Other models tested (one- or three-compartmental models) with non-linear or combined (linear and non-linear) elimination were more complex and did not lead to improved results. The absence of the production of HAHA by patients may strengthen this observation. Indeed, these auto-antibodies are likely to modify the clearance of therapeutic antibodies (Limsakun, 2006; Saito-Yabe *et al*, 2009). The absence of such phenomenon leads to PK parameters similar to endogenous IgG and other antibodies for which no HAHA response has been detected. Indeed, a structural two-compartmental model is commonly reported to describe the behaviour of antibodies. In these models, the associated elimination is mainly linear (Kovarik *et al*, 2001; Bruno *et al*, 2005; Ng *et al*, 2006; Dartois *et al*, 2007) and less frequently non-linear (Mould *et al*, 1999, 2007). Rarely does a combined linear and non-linear process describe the elimination of antibodies (Kuester *et al*, 2008).

Values of the final parameters were similar to those reported for other antibodies, with regard to CL from the central compartment and Q, V1 and V2 (Kovarik *et al*, 2001; Bruno *et al*, 2005; Ng *et al*, 2006; Dartois *et al*, 2007; Mould *et al*, 2007; Kuester *et al*, 2008; Lu *et al*, 2008). This indicates that the HuHMFG1 antibody, similar to other therapeutic antibodies, is mainly distributed in the serum.

Covariate analysis indicated that, despite a weight-based administration in the phase I study, weight was not related to V1 or CL. This indicates that CL seems to remain stable independently of the patient's weight. A similar conclusion was drawn by Ng *et al* (2006) who observed that weight and body surface area were significant covariates, but had a weak impact on patient sample concentrations. Therefore, one could consider recommending a fixed-dose instead of a weight-based dosing regimen for HuHMFG1. Future clinical studies will be necessary to define the optimal dosing regimen.

Interestingly, the hepatic enzyme AST was found to be significantly related to clearance of HuHMFG1. In contrast, in cases in which hepatic enzymes have been investigated as covariates in population PK modelling for other antibodies, they were found not to be significantly relevant (Kuester *et al*, 2008; Lu *et al*, 2008; Newsome and Ernstoff, 2008). Alanine aminotransferase has been found to be strongly linked to another covariate and then eliminated during the removal step of covariate study for trastuzumab (Bruno *et al*, 2005), and ALP was found to be significantly linked to the clearance of pertuzumab (Ng *et al*, 2006). In this study, when only AST was retained during the removal step, ALP and GGT were also found to be linked to the clearance of HuHMFG1 during the first step of covariate study. This is related to the fact that hepatic enzymes were generally elevated together. Retaining only AST during the removal step suggests that AST level alone could partly explain the variability of CL related to hepatic enzymes. Lu *et al* (2008)also observed that AST could be linked to bevacizumab clearance, but found it unlikely that impaired hepatic enzymes would actually alter clearance. Moreover, some authors eliminate covariates because their importance was driven by few patients and simulations showed weak impact on PK parameters (Kuester *et al*, 2008). In our study, the link between AST and clearance was based on data from a significant number of patients, and simulations confirmed the impact of this covariate on HuHMFG1 clearance. We choose to leave AST as a covariate to ensure future investigation into a potentially relevant finding, even if we cannot exclude the fact that the link is due to random chance, as the physiological implication of liver metabolism in IgG clearance is not obvious. A similar strategy was chosen by Ng *et al* (2006), which considered ALP as the covariate of pertuzumab clearance. Data obtained from further studies with HuHMFG1 are required to confirm whether AST is indeed a covariate.

Using population PK parameters, an LSS was developed to facilitate PK sampling and analysis for possible future studies. The retained strategy included both pre- and post-infusion times. In addition, considering the relatively small value of the proportional part of the residual error (18.4%) and the fixed value of the additive part of this error (2.26 mg l^−1^), the selected model presents a reasonably high predictability. Thus, by using Bayesian estimation, the retained LSS was shown to be a powerful tool for assessing the CL of patients with a limited number of samples.

In conclusion, serum concentrations of HuHMFG1 can be successfully described using a two-compartmental model with linear elimination. Pharmacokinetic parameters indicate that the behaviour of this antibody is similar to that of other therapeutic antibodies, especially with regard to a central compartment represented by the serum volume. Interestingly, patient weight was not linked to clearance or central volume of distribution, indicating that a fixed dose might be considered instead of a weight-based dosage, which has been used in the phase II trial with HuHMFG1 in breast cancer. The model presented here offers reasonably high predictability, which, when combined with LSS, could help to guide further PK and pharmacodynamic studies with HuHMFG1. If more data become available linking exposure and efficacy of HuHMFG1, this model would facilitate optimisation of the dose level and dosing schedule.

## Figures and Tables

**Table 1:** Sampling schedule of HuHMFG1

	**First administration (h)**																	
**Posology (mg kg^−1^)**	**Pre-dose**	**EOI** a	**1.5**	**4**	**6**	**12**	**24**	**48**	**72**	**144**	**168**	**216**	**240**	**312**	**336**	**648**	**672**	**840**
1	✓		✓		✓	✓	✓	✓		✓		✓		✓			✓	✓
3^a^			✓		✓	✓	✓	✓		✓		✓		✓		✓		
3^b^	✓	✓		✓	✓	✓	✓		✓		✓		✓	✓	✓	✓		
9	✓	✓		✓	✓	✓	✓		✓	✓								
16	✓	✓		✓	✓	✓	✓		✓	✓								
	**Following administrations**																	
**Posology (mg kg^−1^)**	**Pre-dose**				**EOI**				**Day 3**				**Day 5**			**Day 8**		
1	✓				✓													
3	✓				✓				✓				✓			✓		
9	✓				✓				✓				✓					
16	✓				✓				✓				✓					
	**After the last administration**																	
**Posology (mg kg^−1^)**	**Day 3**		**Day 5**		**Day 7**		**Day 10**		**Day 20**		**Day 28**		**Week 10**		**Week 16**		**Week 22**	
1					✓		✓		✓									
3	✓		✓				✓		✓		✓		✓		✓		✓	
9	✓		✓								✓		✓				✓	
16	✓		✓								✓		✓		✓		✓	

Abbreviation: EOI=end of infusion.

The last administration was undertaken if no toxicity was observed.

a Performed with three patients.

b Performed with six patients.

**Table 2:** Demographic characteristics of covariates in the studied population

**Covariate**	**Median (min**−**max)**
Age (years)	55 (32–72)
Weight (kg)	73.1 (50-108)
Height (cm)	165 (155–179)
Body mass index (kg m^−2^)	25.75 (18.00–40.65)
Total protein concentration (g l^−1^)	68 (54–82)
Albumin concentration (g l^−1^)	41 (27–48)
Creatinine clearance (ml min^−1^)	85.3 (37.2–648.9)
Alkaline phosphatase (Units l^−1^)	86 (47–1127)
Alanine aminotransferase (Units l^−1^)	27 (6–252)
Aspartate aminotransferase (Units l^−1^)	29 (14–1392)
*γ*-Glutamyl transpeptidase (Units l^−1^)	37 (9–1386)
CA15-3	45 (9–16400)
CA27.29	31.3 (3.5–1723)

**Table 3:** Population pharmacokinetic parameters of HuHMFG1 and bootstrap evaluation

	**Final model original data**		**Bootstrap evaluation**	
**Parameter**	**Mean**	**s.d.**	**Mean**	**s.d.**
*V1 (*l*)*				
*θ*	3.31	0.14	3.29	0.13
*ω*^2^	0.042	0.010	0.040	0.010
				
*CL (l h* ^*−1*^ *)*				
*θ*	0.016	0.002	0.016	0.002
*ω*^2^	0.062	0.018	0.059	0.020
				
*Q (l h* ^*−1*^ *)*				
*θ*	0.017	0.004	0.018	0.004
*ω*^2^	NA	NA	NA	NA
				
*V2 (l)*				
*θ*	2.33	0.42	2.32	0.37
*ω*^2^	NA	NA	NA	NA
				
*COV*_*CL*_				
*θ*	0.0036	0.0012	0.0035	0.0012
				
*σ*_*1*_^*2*^ *(proportional)*				
*θ*	0.034	0.004	0.033	0.004
				
*σ*_*2*_^*2*^ *(additive) (mg l*^*−1*^*)*				
*θ*	2.26 (fixed)	NA	2.26 (fixed)	NA

Abbreviations: V1=volume of the central compartment; V2=peripheral volume; Q=inter-compartmental clearance; *θ*=value of the corresponding parameter; *ω*^2^=value of the inter-subject variability associated with the corresponding parameter; COV_CL_=value of the parameter associated with the equation of the covariate; *σ*_1_^2^: proportional part of the residual error; *σ*_2_^2^=additive part of the residual error; NA=not assessable.

**Table 4:** Accuracy (mpe%) and precision (rmse%) of LSSs tested

**No. of samples**	**Sampling time (h)**	**mpe%**	**rmse%**
1	TC	−5.53	10.73
*2*	*TC, EOI*	*−5.68*	*8.59*
2	TC, 4	−5.82	10.60
2	TC, 6	−5.15	10.75
2	TC, 24	−5.24	10.02
3	TC, EOI, 4	−5.58	8.45
3	TC, EOI, 6	−5.45	8.66
8–12	Cycle 1	−9.81	18.68

Abbreviations: mpe%=mean relative prediction error; rmse%=root mean squared relative prediction error; LSS=limited sampling strategy; TC=trough concentration; EOI=end of infusion.

The retained LSS is indicated in italics.

## References

[bib1] Beal SL, Sheiner LB, Boeckmann AJ (eds) (1989–2006) NONMEM users guide (1989–2008). Icon Development Solutions: Ellicott City, MD

[bib2] Brendel K, Comets E, Laffont C, Laveille C, Mentre F (2006) Metrics for external model evaluation with an application to the population pharmacokinetics of gliclazide. Pharm Res 23: 2036–2049 16906454 10.1007/s11095-006-9067-5 PMC2124466

[bib3] Bruno R, Washington CB, Lu JF, Lieberman G, Banken L, Klein P (2005) Population pharmacokinetics of trastuzumab in patients with HER2+ metastatic breast cancer. Cancer Chemother Pharmacol 56: 361–369 15868146 10.1007/s00280-005-1026-z

[bib4] Cockcroft DW, Gault MH (1976) Prediction of creatinine clearance from serum creatinine. Nephron 16: 31–41 1244564 10.1159/000180580

[bib5] Comets E, Brendel K, Mentre F (2008) Computing normalised prediction distribution errors to evaluate nonlinear mixed-effect models: the npde add-on package for R. Comput Methods Programs Biomed 90: 154–166 18215437 10.1016/j.cmpb.2007.12.002

[bib6] Dartois C, Freyer G, Michallet M, Henin E, You B, Darlavoix I, Vermot-Desroches C, Tranchand B, Girard P (2007) Exposure-effect population model of inolimomab, a monoclonal antibody administered in first-line treatment for acute graft-versus-host disease. Clin Pharmacokinet 46: 417–432 17465640 10.2165/00003088-200746050-00004 PMC2760126

[bib7] Holford N, Black P, Couch R, Kennedy J, Briant R (1993a) Theophylline target concentration in severe airways obstruction–10 or 20 mg/l? A randomised concentration-controlled trial. Clin Pharmacokinet 25: 495–505 8119049 10.2165/00003088-199325060-00007

[bib8] Holford N, Hashimoto Y, Sheiner LB (1993b) Time and theophylline concentration help explain the recovery of peak flow following acute airways obstruction. Population analysis of a randomised concentration controlled trial. Clin Pharmacokinet 25: 506–515 8119050 10.2165/00003088-199325060-00008

[bib9] Kovarik JM, Nashan B, Neuhaus P, Clavien PA, Gerbeau C, Hall ML, Korn A (2001) A population pharmacokinetic screen to identify demographic-clinical covariates of basiliximab in liver transplantation. Clin Pharmacol Ther 69: 201–209 11309548 10.1067/mcp.2001.114887

[bib10] Kuester K, Kovar A, Lupfert C, Brockhaus B, Kloft C (2008) Population pharmacokinetic data analysis of three phase I studies of matuzumab, a humanised anti-EGFR monoclonal antibody in clinical cancer development. Br J Cancer 98: 900–906 18319714 10.1038/sj.bjc.6604265 PMC2266843

[bib11] Kumle M (2008) Declining breast cancer incidence and decreased HRT use. Lancet 372: 608–610 18722851 10.1016/S0140-6736(08)61255-6

[bib12] Limsakun T (2006) Immunogenicity. In Clinical Pharmacology of Therapeutic Proteins Mahmood I (ed) pp. 197–227. Pine House: Rockville

[bib13] Lu JF, Bruno R, Eppler S, Novotny W, Lum B, Gaudreault J (2008) Clinical pharmacokinetics of bevacizumab in patients with solid tumors. Cancer Chemother Pharmacol 62: 779–786 18205003 10.1007/s00280-007-0664-8

[bib14] Mould DR, Baumann A, Kuhlmann J, Keating MJ, Weitman S, Hillmen P, Brettman LR, Reif S, Bonate PL (2007) Population pharmacokinetics-pharmacodynamics of alemtuzumab (Campath) in patients with chronic lymphocytic leukaemia and its link to treatment response. Br J Clin Pharmacol 64: 278–291 17506867 10.1111/j.1365-2125.2007.02914.x PMC2000651

[bib15] Mould DR, Davis CB, Minthorn EA, Kwok DC, Elliott MJ, Luggen ME, Totoritis MC (1999) A population pharmacokinetic-pharmacodynamic analysis of single doses of clenoliximab in patients with rheumatoid arthritis. Clin Pharmacol Ther 66: 246–257 10511060 10.1016/S0009-9236(99)70032-9

[bib16] Newsome BW, Ernstoff MS (2008) The clinical pharmacology of therapeutic monoclonal antibodies in the treatment of malignancy; have the magic bullets arrived? Br J Clin Pharmacol 66: 6–19 18503606 10.1111/j.1365-2125.2008.03187.x PMC2485255

[bib17] Ng CM, Lum BL, Gimenez V, Kelsey S, Allison D (2006) Rationale for fixed dosing of pertuzumab in cancer patients based on population pharmacokinetic analysis. Pharm Res 23: 1275–1284 16715358 10.1007/s11095-006-0205-x

[bib18] Pegram MD, Borges VF, Ibrahim N, Fuloria J, Shapiro C, Perez S, Wang K, Schaedli Stark F, Courtenay Luck N (2009) Phase I dose escalation pharmacokinetic assessment of intravenous humanized anti-MUC1 antibody AS1402 in patients with advanced breast cancer. Breast Cancer Res 11: R73 19811637 10.1186/bcr2409 PMC2790853

[bib19] Pericleous LM, Richards J, Epenetos AA, Courtenay-Luck N, Deonarain MP (2005) Characterisation and internalisation of recombinant humanised HMFG-1 antibodies against MUC1. Br J Cancer 93: 1257–1266 16265351 10.1038/sj.bjc.6602847 PMC3216111

[bib20] Royer B, Jullien V, Guardiola E, Heyd B, Chauffert B, Kantelip JP, Pivot X (2009) Population pharmacokinetics and dosing recommendations for cisplatin during intraperitoneal peroperative administration: development of a limited sampling strategy for toxicity risk assessment. Clin Pharmacokinet 48: 169–180 19385710 10.2165/00003088-200948030-00003

[bib21] Saito-Yabe M, Yoshigae Y, Takasaki W, Kurihara A, Ikeda T, Okazaki O (2009) Highly frequent anti-idiotype antibody in cynomolgus monkeys developed against mouse-derived regions of anti-Fas antibody humanized by complementarity determining region grafting. Br J Pharmacol 158: 548–557 19645714 10.1111/j.1476-5381.2009.00326.x PMC2757695

[bib22] Sheiner LB (1997) Learning versus confirming in clinical drug development. Clin Pharmacol Ther 61: 275–291 9084453 10.1016/S0009-9236(97)90160-0

[bib23] Singh R, Bandyopadhyay D (2007) MUC1: a target molecule for cancer therapy. Cancer Biol Ther 6: 481–486 18027437 10.4161/cbt.6.4.4201

[bib24] Snijdewint FG, von Mensdorff-Pouilly S, Karuntu-Wanamarta AH, Verstraeten AA, Livingston PO, Hilgers J, Kenemans P (2001) Antibody-dependent cell-mediated cytotoxicity can be induced by MUC1 peptide vaccination of breast cancer patients. Int J Cancer 93: 97–106 11391628 10.1002/ijc.1286

